# Effect of social odor context on the emission of isolation-induced ultrasonic vocalizations in the BTBR T+tf/J mouse model for autism

**DOI:** 10.3389/fnins.2015.00073

**Published:** 2015-03-18

**Authors:** Markus Wöhr

**Affiliations:** Behavioral Neuroscience, Experimental and Physiological Psychology, Philipps-University of MarburgMarburg, Germany

**Keywords:** animal model, neurodevelopmental disorder, ultrasonic vocalization, ultrasonic communication, maternal odor

## Abstract

An important diagnostic criterion for social communication deficits in autism spectrum disorders (ASD) are difficulties in adjusting behavior to suit different social contexts. While the BTBR T+tf/J (BTBR) inbred strain of mice is one of the most commonly used mouse models for ASD, little is known about whether BTBR mice display deficits in detecting changes in social context and their ability to adjust to them. Here, it was tested therefore whether the emission of isolation-induced ultrasonic vocalizations (USV) in BTBR mouse pups is affected by the social odor context, in comparison to the standard control strain with high sociability, C57BL/6J (B6). It is known that the presence of odors from mothers and littermates leads to a calming of the isolated mouse pup, and hence to a reduction in isolation-induced USV emission. In accordance with their behavioral phenotypes with relevance to all diagnostic core symptoms of ASD, it was predicted that BTBR mouse pups would not display a calming response when tested under soiled bedding conditions with home cage bedding material containing maternal odors, and that similar isolation-induced USV emission rates would be seen in BTBR mice tested under clean and soiled bedding conditions. Unexpectedly, however, the present findings show that BTBR mouse pups display such a calming response and emit fewer isolation-induced USV when tested under soiled as compared to clean bedding conditions, similar to B6 mouse pups. Yet, in contrast to B6 mouse pups, which emitted isolation-induced USV with shorter call durations and lower levels of frequency modulation under soiled bedding conditions, social odor context had no effect on acoustic call features in BTBR mouse pups. This indicates that the BTBR mouse model for ASD does not display deficits in detecting changes in social context, but has a limited ability and/or reduced motivation to adjust to them.

## Introduction

The BTBR T+tf/J (BTBR) inbred strain of mice is one of the most commonly used mouse models for autism spectrum disorders (ASD). BTBR mice display behavioral phenotypes with relevance to all diagnostic core symptoms of ASD, namely persistent deficits in reciprocal social interaction and communication across multiple contexts, together with restricted, repetitive patterns of behavior, activities, and interests (DSM-5, 2013), as compared to the social mouse strain C57BL/6J (B6; for review see: Blanchard et al., 2012; Meyza et al., 2013; Careaga et al., 2015).

Specifically, BTBR mice display reduced reciprocal social interaction behavior as juveniles (Yang et al., 2007b, 2009; McFarlane et al., 2008; Jones-Davis et al., 2013) and lack of sociability as adults (Bolivar et al., 2007; Moy et al., 2007; Yang et al., 2007a,b, 2009; McFarlane et al., 2008; Defensor et al., 2011; Jones-Davis et al., 2013) in standard laboratory settings, but also semi-natural environments (Pobbe et al., 2010), possibly due to reduced social motivation (Pearson et al., 2012; Martin et al., 2014). For assessing communication deficits, ultrasonic vocalizations (USV) are typically studied. As pups, BTBR mice display an unusual pattern of USV categories, including high levels of harmonics, two-syllable, and composite calls, but vocalize more than B6 mice when being isolated from mother and littermates (Scattoni et al., 2008). During adolescence, low emission rates of pro-social USV were observed in BTBR mice, consistent with their strongly reduced juvenile reciprocal social interaction behavior (Scattoni et al., 2013). Likewise, during reciprocal social interactions in adulthood also low emission rates of pro-social USV were obtained (Scattoni et al., 2011; Yang et al., 2013). Moreover, male BTBR mice do not emit USV to attract females and display reduced scent marking behavior in response to female urine cues, in stark contrast to male B6 mice (Wöhr et al., 2011b), while scent marking behavior in response to male urine cues was found to be unchanged (Roullet et al., 2011). Deficits in the social transmission of food preferences were also reported (McFarlane et al., 2008). Finally, BTBR mice show high levels of restricted, repetitive behavior, such as perseverative self-grooming and marble-burying (McFarlane et al., 2008; Yang et al., 2009; Pobbe et al., 2010; Pearson et al., 2011; Amodeo et al., 2012; Jones-Davis et al., 2013; Molenhuis et al., 2014) or altered exploratory behavior in the hole board task (Moy et al., 2008) and the repetitive novel object contact task (Pearson et al., 2011). They were also reported to display deficits in reversal learning in the Morris water maze (Moy et al., 2007; Yang et al., 2012a) and a set-shifting task (Molenhuis et al., 2014), yet conflicting results were obtained in T-maze reversal learning and related tasks (Moy et al., 2007; Amodeo et al., 2012; Guariglia and Chadman, 2013).

In addition, BTBR mice are characterized by alterations in brain development and morphology associated with ASD, including a lack of the corpus callosum (Wahlsten et al., 2003; Kusek et al., 2007; MacPherson et al., 2008; Jones-Davis et al., 2013), altered functional connectivity networks (Dodero et al., 2013; Ellegood et al., 2013, 2015; Miller et al., 2013; Gogolla et al., 2014; Sforazzini et al., 2015), as well as reduced hippocampal neurogenesis and changes in neurodevelopmental proteins (Stephenson et al., 2011). BTBR mice further display ASD-related alterations in neurotransmitter systems, including serotonin (Gould et al., 2011, 2014; Zhang et al., 2014), dopamine (Squillace et al., 2014), and acetylcholine (McTighe et al., 2013), as well as in neuromodulators, such as oxytocin (Silverman et al., 2010b) and endocannabinoids (Liu et al., 2009; Onaivi et al., 2011; Gould et al., 2014). Persistent immune dysregulation was also reported (Heo et al., 2011; Onore et al., 2013; Schwartzer et al., 2013; Zhang et al., 2013). Not surprisingly, the BTBR inbred strain of mice is therefore a mouse model for ASD that is commonly used to test new pharmacological compounds and strategies for their efficacy in reversing ASD-related behavioral phenotypes, such as negative allosteric modulation of the mGluR5 receptor (Silverman et al., 2012), long-term exposure to intranasal oxytocin (Bales et al., 2014), and others (Silverman et al., 2010a, 2013a,b; Burket et al., 2013, 2014; Amodeo et al., 2014a,b; Han et al., 2014; Karvat and Kimchi, 2014; Langley et al., 2015).

An important diagnostic criterion for social communication deficits in ASD are difficulties in adjusting behavior to suit different social contexts (DSM-5, 2013). However, little is known about whether the BTBR mouse model for ASD displays deficits in detecting changes in social context and their ability to adjust to them. Yet, the fact that the strain of the partner during reciprocal social interactions was reported to have minimal effects on the social behavioral repertoire displayed by BTBR mice is in stark contrast to the changes that were observed in B6 mice (Yang et al., 2012a) and suggests that BTBR mice have difficulties in adjusting their behavior to different social contexts. Here, it was tested therefore whether the emission of isolation-induced USV in BTBR mouse pups is affected by the social odor context, in comparison to the standard control strain with high sociability, B6. It is known that the presence of odors from mothers and littermates leads to a calming of the isolated mouse pup, and hence to a reduction in isolation-induced USV emission (Branchi et al., 1998; Marchlewska-Koj et al., 1999; Kapusta and Szentgyörgyi, 2004; Moles et al., 2004; Zanettini et al., 2010; for similar findings in voles and rats see: Oswalt and Meier, 1975; Conely and Bell, 1978; Kapusta et al., 1995; Szentgyörgyi et al., 2008; but see: Lemasson et al., 2005). Highlighting the relevance of this calming response for behavioral phenotyping of mouse models for ASD, it was further shown that μ-opioid deficient mice do not display a reduction in isolation-induced USV emission rates in the presence of odors from mothers and littermates (Moles et al., 2004), consistent with a variety of other social and communication deficits displayed by this ASD mouse model (Tian et al., 1997; Wöhr et al., 2011a; Cinque et al., 2012; Becker et al., 2014; Gigliucci et al., 2014; for review see: Oddi et al., 2013).

In accordance with their behavioral phenotypes with relevance to all diagnostic core symptoms of ASD, it was predicted that BTBR mouse pups would not display a calming response when tested under soiled bedding conditions with home cage bedding material containing maternal odors, and that similar isolation-induced USV emission rates would be seen in BTBR mice tested under clean and soiled bedding conditions, while lower isolation-induced USV emission rates would occur in B6 mice tested under soiled bedding conditions as compared to clean bedding conditions.

## Materials and methods

### Animals and housing

Subject mice were *N* = 30 BTBR T+tf/J (BTBR) and *N* = 30 C57BL/6J (B6) mice. Breeding pairs were purchased from The Jackson Laboratory (Bar Harbor, ME, USA) and bred at the National Institute of Mental Health in Bethesda, MD, USA. About 2 weeks after pairing for breeding, females were individually housed and subsequently inspected daily for pregnancy and delivery. The day of birth was considered as postnatal day (PND) 0. All mice were housed in polycarbonate Makrolon cages (369 × 156 × 132 mm, 435 cm^2^; 1145T; Tecniplast, Milan, Italy). Bedding, paper strips, a nestlet square, and a cardboard tube were provided in each cage. Standard rodent chow and water were available *ad libitum*. The colony room was maintained on a 12:12 light/dark cycle with lights on at 06:00 h, at 20°C temperature and 55% humidity. All procedures were conducted in strict compliance with the National Institutes of Health Guidelines for the Care and Use of Laboratory Animals and approved by the National Institute of Mental Health Animal Care and Use Committee.

### Social odor context manipulation

An experimental design with two independent factors was used in order to study the effects of social odor context on isolation-induced pup USV in a strain-dependent manner, namely strain (BTBR vs. B6) and social odor context (clean bedding vs. soiled bedding), with *N* = 15 per strain and social odor context. To this aim, mouse pups from four different litters per strain (litter size: BTBR: 7.50 ± 1.50; B6: 7.50 ± 1.26; typically with a male: female ratio of approximately 50:50 in both strains) were tested on PND8, using either clean bedding or soiled bedding from the home cage. Random group assignment was used, with approximately 50% of pups per sex from a given litter being tested under clean bedding or soiled bedding conditions, respectively. Pups were tested only once to avoid carry over effects. Home cages used to obtain soiled bedding material were not cleaned for at least 2 days prior testing in order to expose mouse pups to sufficiently distinct odor stimuli.

### Isolation-induced USV—recording

Pups were isolated from their mother and littermates on PND8 for 5 min under room temperature (22–24°C; humidity: 3–55%). Pups were removed individually from the nest at random and gently placed into an isolation container made of glass (10 × 8 × 7 cm; open surface), containing either clean bedding or soiled bedding depending on experimental group. The isolation container was surrounded by a sound attenuating box (18 × 18 × 18 cm) made of Styrofoam (thickness of walls: 4 cm). USV emission was monitored by an UltraSoundGate Condenser Microphone CM16 (Avisoft Bioacoustics, Berlin, Germany) placed in the roof of the sound attenuating box, 10 cm above the floor. The microphone was connected via an UltraSoundGate 116 USB audio device (Avisoft Bioacoustics) to a personal computer, where acoustic data were recorded with a sampling rate of 250,000 Hz in 16 bit format by Avisoft RECORDER (version 2.97; Avisoft Bioacoustics). The microphone was sensitive to frequencies of 15–180 kHz with a flat frequency response (±6 dB) between 25 and 140 kHz. After the 5 min isolation period, body weight and body temperature were determined. Body weight was measured using a palmscale (PS6-250; My Weigh Europe, Hückelhoven, Germany). For body temperature determination a DiGiSense Thermistor Thermometer (Thermo Fisher Scientific Inc., Waltham, MA, USA) was used. Body temperature was measured by gentle application of the thermal probe onto the stomach of the mouse pup for 20 s. Isolation occurred between 8:00 and 12:00 h during the light phase of the 12:12 h light/dark cycle. Prior to each test, behavioral equipment was cleaned using a 70% ethanol solution, followed by water, and dried with paper towels.

### Isolation-induced USV—analysis

For acoustical analysis, recordings were transferred to Avisoft SASLab Pro (version 4.50; Avisoft Bioacoustics) and a fast Fourier transform was conducted (512 FFT length, 100% frame, Hamming window, and 75% time window overlap), resulting in spectrograms with 488 Hz of frequency resolution and 0.512 ms of time resolution. Detection of isolation-induced USV was provided by an automatic threshold-based algorithm (amplitude threshold: −40 dB) and a hold-time mechanism (hold time: 10 ms). Since no USV were detected below 30 kHz, a high-pass filter of 30 kHz was used to reduce background noise outside the relevant frequency band to 0 dB. The accuracy of USV detection by the software was verified manually by an experienced user. When necessary, missed USV were marked by hand to be included in the automatic parameter analysis. Total number of isolation-induced USV was calculated for the entire 5 min recording session. Based on previous studies on isolation-induced USV in mouse pups (Wöhr et al., 2008, 2011b; Kurz et al., 2010; Yang et al., 2012b), the following additional parameters were included: latency to start calling, total calling time, call duration, peak frequency, peak amplitude, and frequency modulation. Peak frequency and peak amplitude were derived from the average spectrum of the entire USV. Peak amplitude, i.e., loudness, was defined as the point with the highest energy within the spectrum. Peak frequency was defined as the frequency at the location of the peak amplitude within the spectrum. The extent of frequency modulation was defined as the difference between the lowest and the highest peak frequency within each USV. In addition, USV subtypes were determined by means of density blots (Wöhr, 2014), depicting call duration and frequency modulation. Finally, to assess the temporal organization of isolation-induced USV emission, sequential analyses were performed by correlating the durations of given isolation-induced USV with the durations of the previous ones (N-1), the ones two before (N-2), and the ones three before (N-3), as described before (Wöhr, 2014).

### Statistical analysis

For statistical comparisons, Two-Way ANOVAs with the between-subject factors strain (BTBR vs. B6) and social odor context (clean bedding vs. soiled bedding) were calculated, followed by unpaired *t*-tests when appropriate. Pearson's product moment statistics were used to run correlation analyses between the durations of given isolation-induced USV with the durations of the previous ones (N-1), the ones two before (N-2), and the ones three before (N-3) in mouse pups that emitted >3 isolation-induced USV. Paired *t*-tests were used to compare correlation coefficients against chance level. Sex had no effect on the emission of isolation-induced USV (all *p* > 0.100). A *p*-value of < 0.050 was considered statistically significant.

## Results

### Effects of social odor context on isolation-induced USV in BTBR and B6 mouse pups

**Figure 1:**
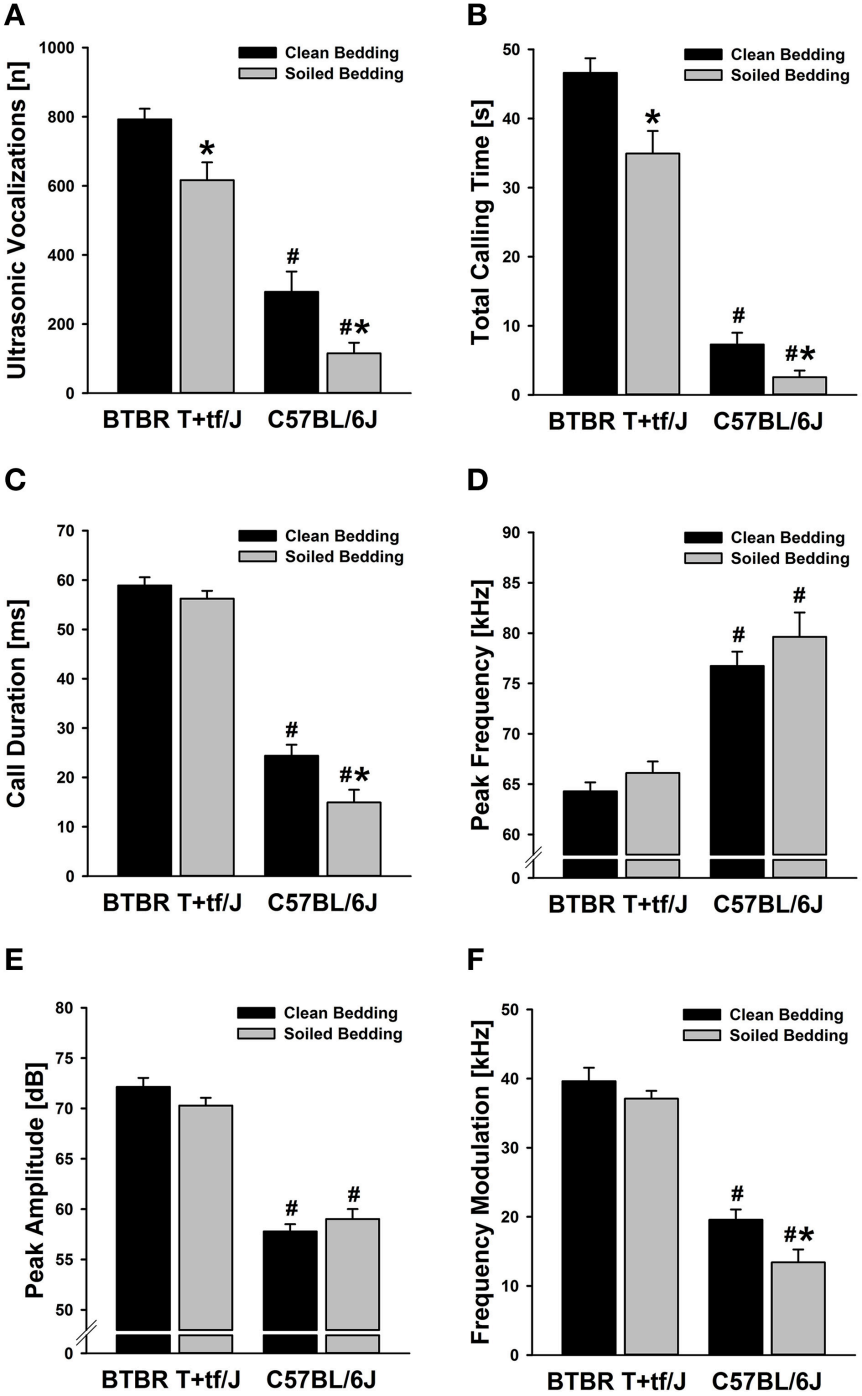
**Effects of social odor context on isolation-induced ultrasonic vocalizations (USV) emitted by BTBR T+tf/J (left) and C57BL/6J (right) mouse pups (*N* = 15 per strain and social odor context)**. **(A)** Total number of isolation-induced USV [n] and **(B)** total calling time in seconds [s] in BTBR T+tf/J and C57BL/6J mouse pups tested under clean (black) and soiled (gray) bedding conditions. **(C)** Call duration in milliseconds [ms], **(D)** peak frequency in kilohertz [kHz], **(E)** peak amplitude in decibel [dB], and **(F)** frequency modulation in kilohertz [kHz] of isolation-induced USV emitted by BTBR T+tf/J and C57BL/6J mouse pups tested under clean (black) and soiled (gray) bedding conditions. Data are presented as means ± standard errors of the mean. ^*^p < 0.050 for soiled bedding vs. clean bedding; ^#^*p* < 0.050 for BTBR T+tf/J vs. C57BL/6J mouse pups.

**Figure 2:**
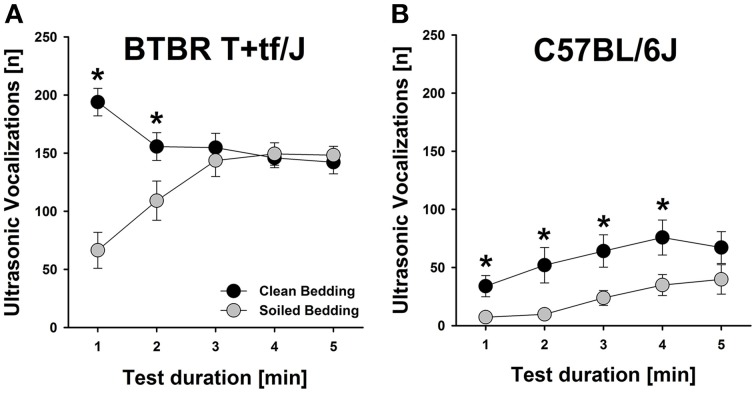
**Effects of social odor context on isolation-induced ultrasonic vocalizations (USV) emitted by BTBR T+tf/J (left) and C57BL/6J (right) mouse pups (*N* = 15 per strain and social odor context)—Time course**. Number of isolation-induced USV [n] in **(A)** BTBR T+tf/J and **(B)** C57BL/6J mouse pups tested under clean (black) and soiled (gray) bedding conditions per minute [min]. Data are presented as means ± standard errors of the mean. ^*^p < 0.050 for soiled bedding vs. clean bedding.

Social odor context significantly affected the emission of isolation-induced USV [main effect social odor context: *F*_(1, 56)_ = 15.646, *p* < 0.001; Figure 1A], with emission rates significantly differing between strains [main effect strain: *F*_(1, 56)_ = 124.807, *p* < 0.001; interaction social odor context × strain: *F*_(1, 56)_ = 0.001, *p* = 0.983; Figure 1A]. Specifically, both, BTBR and B6 mouse pups tested under home cage bedding conditions emitted significantly fewer isolation-induced USV than littermates tested under clean cage bedding conditions [*t*_(28)_ = 2.939, *p* = 0.007 and *t*_(28)_ = 2.664, *p* = 0.013; respectively]. In BTBR mouse pups, a significant reduction in isolation-induced USV was seen in the first 2 min of testing [min 1–5: *t*_(28)_ = 6.557, *p* < 0.001; *t*_(28)_ = 2.255, *p* = 0.032; *t*_(28)_ = 0.605, *p* = 0.550; *t*_(28)_ = 0.270, *p* = 0.789; *t*_(28)_ = 0.482, *p* = 0.633; respectively; Figure 2A], whereas in B6 mouse pups significant reductions were seen in the first 4 min of testing [min 1–5; *t*_(28)_ = 2.835, *p* = 0.008; *t*_(28)_ = 2.703, *p* = 0.012; *t*_(28)_ = 2.638, *p* = 0.013; *t*_(28)_ = 2.332, *p* = 0.027; *t*_(28)_ = 1.470, *p* = 0.153; respectively; Figure 2B]. As expected, BTBR mouse pups emitted significantly more isolation-induced USV than B6 mouse pups under clean bedding conditions [*t*_(28)_ = 7.487, *p* < 0.001], and in line with the results obtained under clean bedding conditions, BTBR mouse pups also emitted significantly more isolation-induced USV than B6 mouse pups under soiled bedding conditions [*t*_(28)_ = 8.385, *p* < 0.001].

Consistently, total calling time was significantly lower in mouse pups tested in soiled bedding than in littermates tested in clean bedding [main effect social odor context: *F*_(1, 56)_ = 14.127, *p* < 0.001; Figure 1B], with total calling times significantly differing between strains [main effect strain: *F*_(1, 56)_ = 269.882, *p* < 0.001; interaction social odor context × strain: *F*_(1, 56)_ = 2.536, *p* = 0.117; Figure 1B]. Importantly, however, the reduction was again seen in both, BTBR and B6 mouse pups [*t*_(28)_ = 2.996, *p* = 0.006 and *t*_(28)_ = 2.408, *p* = 0.023; respectively], despite total calling times being significantly higher in BTBR than in B6 mouse pups under clean and soiled bedding conditions [*t*_(28)_ = 14.396, *p* < 0.001 and *t*_(28)_ = 9.511, *p* < 0.001; respectively]. The reductions in isolation-induced USV emission rates and total calling times seen in mouse pups tested in a soiled odor context were not due to longer latencies to start calling [main effect social odor context: *F*_(1, 56)_ = 1.085, *p* = 0.302; not shown], yet latencies to start calling differed significantly between strains [main effect strain: *F*_(1, 56)_ = 9.654, *p* = 0.003; interaction social odor context × strain: *F*_(1, 56)_ = 0.061, *p* = 0.805; not shown]. Specifically, the latency to start calling was significantly reduced in BTBR mouse pups as compared to B6 mouse pups under both, clean and soiled bedding conditions [*t*_(28)_ = 2.110, *p* = 0.044 and *t*_(28)_ = 2.281, *p* = 0.030; respectively], consistent with an overall higher level of isolation-induced USV emission in BTBR mouse pups.

Importantly, two out of four acoustic call features determined were also significantly affected by social odor context, namely mean call duration [main effect social odor context: *F*_(1, 54)_ = 8.937, *p* = 0.004; Figure 1C] and frequency modulation [main effect social odor context: *F*_(1, 54)_ = 6.927, *p* = 0.011; Figure 1F], with both of them also significantly differing between strains [main effect strain: *F*_(1, 54)_ = 349.882, *p* < 0.001 and *F*_(1, 54)_ = 175.969, *p* < 0.001; respectively; interaction social odor context × strain: *F*_(1, 54)_ = 2.817, *p* = 0.099 and *F*_(1, 54)_ = 1.221, *p* = 0.274; respectively; Figures 1C,F]. Interestingly, however, when comparing BTBR mouse pups tested in the two social odor contexts, there were no significant differences in mean call duration [*t*_(28)_ = 1.152, *p* = 0.259] and frequency modulation [*t*_(28)_ = 1.116, *p* = 0.274]. In contrast to BTBR mouse pups, mean call duration was affected by social odor context in B6 mouse pups, which emitted significantly shorter isolation-induced USV when tested in soiled bedding than littermates tested in clean bedding [*t*_(26)_ = 2.789, *p* = 0.010]. Furthermore, frequency modulation was affected by social odor context, with B6 mouse pups tested in soiled bedding emitting significantly less frequency-modulated isolation-induced USV than littermates tested in clean bedding [*t*_(26)_ = 2.556, *p* = 0.017], again in contrast to BTBR mouse pups. Under both, clean and soiled bedding conditions, isolation-induced USV emitted by BTBR mouse pups were significantly longer [*t*_(28)_ = 12.464, *p* < 0.001 and *t*_(28)_ = 9.511, *p* < 0.001; respectively] and higher in frequency modulation [*t*_(28)_ = 12.373, *p* < 0.001 and *t*_(28)_ = 10.964, *p* < 0.001; respectively] when compared to isolation-induced USV emitted by B6 mouse pups.

Finally, however, the two other acoustic call features determined were not significantly affected by social odor context, namely peak frequency [main effect social odor context: *F*_(1, 54)_ = 2.324, *p* = 0.133; Figure 1D] and peak amplitude [main effect social odor context: *F*_(1, 54)_ = 0.130, *p* = 0.719; Figure 1E], yet significant differences between strains were detected for both measures [main effect strain: *F*_(1, 54)_ = 070.351, *p* < 0.001 and *F*_(1, 54)_ = 228.079, *p* < 0.001; respectively; interaction social odor context × strain: *F*_(1, 54)_ = 0.123, *p* = 0.727 and *F*_(1, 54)_ = 3.329, *p* = 0.074; respectively; Figures 1D,E]. Specifically, under both, clean and soiled bedding conditions, isolation-induced USV emitted by BTBR mouse pups were significantly lower in peak frequency [*t*_(28)_ = 8.050, *p* < 0.001 and *t*_(28)_ = 5.172, *p* < 0.001; respectively], but higher in peak amplitude [*t*_(28)_ = 12.373, *p* < 0.001 and *t*_(28)_ = 9.101, *p* < 0.001; respectively] when compared to isolation-induced USV emitted by B6 mouse pups.

Of note, body weight and temperature did not differ significantly between the two social odor contexts [main effect social odor context: *F*_(1, 56)_ = 0.066, *p* = 0.797 and *F*_(1, 56)_ = 0.003, *p* = 0.954; respectively; not shown], yet significant differences between strains were detected for both measures [main effect strain: *F*_(1,56)_ = 40.815, *p* < 0.001 and *F*_(1, 56)_ = 6.110, *p* = 0.017; respectively; interaction social odor context × strain: *F*_(1, 56)_ = 0.007, *p* = 0.932 and *F*_(1, 56)_ = 0.001, *p* = 0.995; respectively; not shown]. Specifically, as expected, body weight was significantly higher in BTBR than in B6 mouse pups under clean and soiled bedding conditions [*t*_(28)_ = 4.053, *p* < 0.001 and *t*_(28)_ = 5.147, *p* < 0.001; respectively]. Yet, when comparing strains tested either in clean or soiled bedding, no significant differences in body temperature were detected, but BTBR tended to have higher body temperatures than B6 [*t*_(28)_ = 1.748, *p* = 0.091 and *t*_(28)_ = 1.748, *p* = 0.091; respectively].

### Detailed spectrographic analysis—call clustering and temporal organization in BTBR and B6 mouse pups

**Figure 3:**
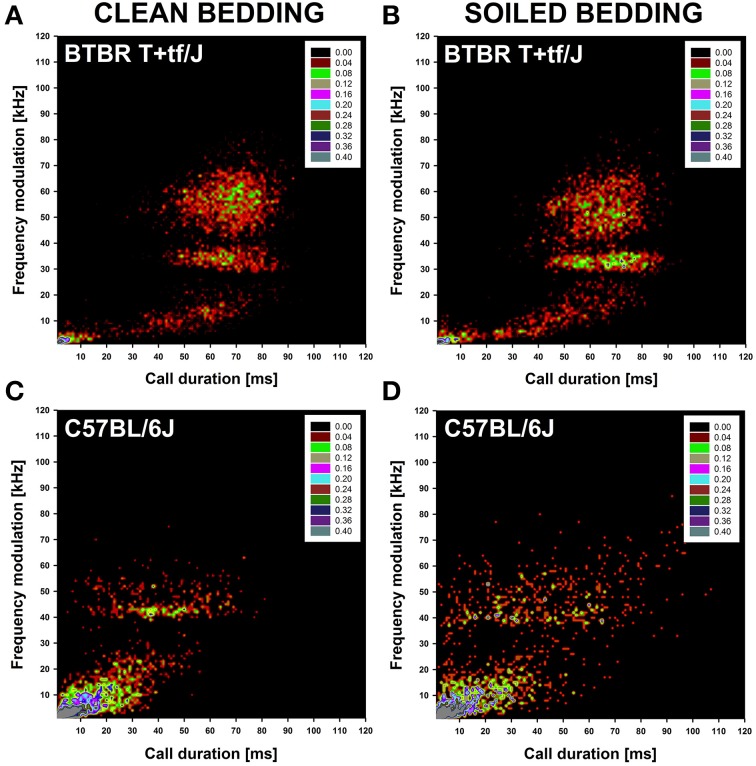
**Distribution of individual isolation-induced ultrasonic vocalizations (USV) in BTBR T+tf/J (upper panel) and C57BL/6J (lower panel) mouse pups (*N* = 15 per strain and social odor context)**. Density plots depicting the distribution of individual isolation-induced USV depending on call duration in milliseconds [ms] and frequency modulation in kilohertz [kHz] in BTBR T+tf/J mouse pups tested under **(A)** clean and **(B)** soiled bedding conditions and in C57BL/6J mouse pups tested under **(C)** clean and **(D)** soiled bedding conditions. Color coding reflects frequencies as percentages.

A more detailed analysis was performed to identify clusters of isolation-induced USV emitted by BTBR and B6 mouse pups under clean and soiled bedding conditions by means of density plots. For generating density plots, the two acoustic call features most strongly affected by social odor context were used, namely call duration and frequency modulation. In BTBR mouse pups tested under clean bedding conditions, four prominent call clusters were detected. One cluster was characterized by short call durations (<10 ms) and low levels of frequency modulation (<10 kHz). The other three clusters were characterized by long call durations (30–90 ms), with varying levels of frequency modulation, namely low (<20 kHz), moderate (30–40 kHz), and high (40–70 kHz; Figure 3A). Consistent with the lack of significant differences between clean and soiled bedding conditions in mean call duration and frequency modulation in BTBR mouse pups, the social odor context had only minor effects on call clustering, with the call clusters characterized by long call durations (30–90 ms) and low (<20 kHz) or moderate (30–40 kHz) levels of frequency modulation being more prominent (Figure 3B). While there were only minor social odor context effects on call clustering in BTBR mouse pups, call clustering markedly differed between BTBR and B6 mouse pups. Whereas in BTBR four prominent call clusters were detected, only two prominent call clusters were detected in B6 mouse pups. One cluster was characterized by short call durations (<40 ms) and low levels of frequency modulation (<30 kHz) and therefore broader than the corresponding call cluster in BTBR mouse pups. The second one was characterized by comparably long call durations (30–70 ms) and moderate levels of frequency modulation (40–50 kHz), and thus possibly corresponding to the call cluster in BTBR that was characterized by long call durations (30–90 ms) and moderate frequency modulation (30–40 kHz; Figure 3C). In B6 mouse pups, social odor context affected call clustering, with the second call cluster characterized by comparably long call durations (30–70 ms) and moderate levels of frequency modulation (40–50 kHz) being less prominent and coherent under soiled bedding conditions, in line with the overall reduced mean call duration and frequency modulation (Figure 3D).

**Figure 4:**
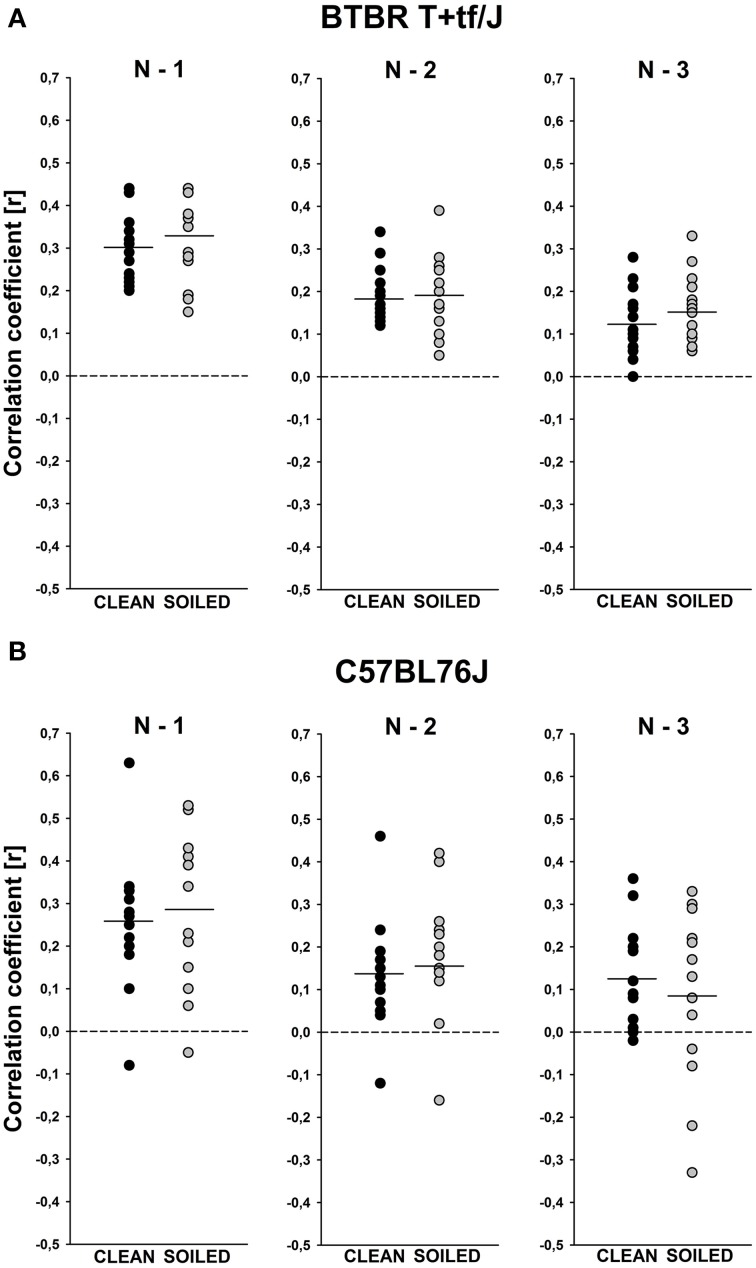
**Sequential analysis of the durations of subsequent isolation-induced ultrasonic vocalizations (USV) indicating non-random call emission patterns in BTBR T+tf/J (upper panel) and C57BL/6J (lower panel) mouse pups (*N* = 15 per strain and social odor context)**. Correlations between the call durations of given isolation-induced USV and the call durations of the previous ones (N–1), the call durations of the ones two before (N–2), or the call durations of the ones three before (N–3) for **(A)** BTBR T+tf/J and **(B)** C57BL/6J mouse pups tested under clean (black circles) and soiled (gray circles) bedding conditions. Each circle represents one mouse pup that emitted >3 isolation-induced USV. The solid line represents the mean per condition.

An additional sequential analysis of the durations of subsequent isolation-induced USV finally indicated that the call emission pattern is not random in BTBR mouse pups tested under both, clean and soiled, bedding conditions, since the durations of given isolation-induced USV could be predicted by the durations of the previous ones [N–1; clean bedding: *t*_(14)_ = 15.248, *p* < 0.001; soiled bedding: *t*_(14)_ = 12.774, *p* < 0.001], by the ones two before [N–2; clean bedding: *t*_(14)_ = 10.497, *p* < 0.001; soiled bedding: *t*_(14)_ = 8.022, *p* < 0.001], and by the ones three before [N–3; clean bedding: *t*_(14)_ = 6.353, *p* < 0.001; soiled bedding: *t*_(14)_ = 7.313, *p* < 0.001; Figure 4A]. Evidence for such a non-random call emission pattern was also obtained in B6 mouse pups, again, under both, clean and soiled, bedding conditions [N–1; clean bedding: *t*_(13)_ = 6.255, *p* < 0.001; soiled bedding: *t*_(12)_ = 5.647, *p* < 0.001; N–2; clean bedding: *t*_(13)_ = 3.970, *p* = 0.002; soiled bedding: *t*_(12)_ = 3.187, *p* = 0.008; N–3; clean bedding: *t*_(13)_ = 3.992, *p* = 0.002; soiled bedding: *t*_(12)_ = 1.479, *p* = 0.165; Figure 4B]. Correlation coefficients did not differ between BTBR and B6 or between clean and soiled bedding conditions (all *p* > 0.100).

## Discussion

An important diagnostic criterion for social communication deficits in ASD are difficulties in adjusting behavior to suit different social contexts (DSM-5, 2013). In experimental studies assessing social context effects on social behavior, for instance, individuals with ASD display insensitivity to social reputation as assessed by the occurrence of charitable donations in the presence or absence of an observer (Izuma et al., 2011; Cage et al., 2013) or flattery behavior following rating of pictures depending on the drawer's presence (Chevallier et al., 2012b). Individuals with ASD further show resistance to social pressure in the Asch conformity experiment (Bowler and Worley, 1994; Yafai et al., 2014), more fixed strategies disregarding the partner's beliefs in a social hunting game (Yoshida et al., 2010) or trustworthiness in an economic trust game (Ewing et al., 2015), and lack of social gaze influences on motor action control (Schilbach et al., 2012). Also, in a study focusing on social communication, individuals with ASD were found to use attention-directing behavior less frequently than controls and their behavior varied less across social contexts (Landry and Loveland, 1989). Together, these experimental findings echo anecdotal reports of parents emphasizing that individuals with ASD seem only mildly influenced by considerations of impression management (for review see: Chevallier et al., 2012a). Finally, social context appears to have opposite effects on the occurrence of repetitive patterns of behavior in healthy human subjects and individuals with ASD. While in healthy human subjects repetitive behavior is inhibited in social situations (Asendorpf, 1980), studies in individuals with ASD indicate that repetitive behavior is unchanged or even increased when exposed to a social context (Baron-Cohen, 1989; Carruthers, 1996).

Considering the diagnostic criteria for ASD and the experimental findings obtained in human ASD studies, surprisingly little is known about difficulties in adjusting behavior to suit different social contexts in mouse models for ASD (Wöhr and Scattoni, 2013). Even in the BTBR mouse model for ASD, which is one of the most commonly used mouse models (for review see: Blanchard et al., 2012; Meyza et al., 2013; Careaga et al., 2015), no study explicitly addressed this issue so far. The present findings show for the first time that BTBR mouse pups adjust their emission of isolation-induced USV to different social contexts. Specifically, they displayed a calming response and emitted fewer isolation-induced USV when tested under soiled bedding conditions with home cage bedding material containing maternal odors as compared to clean bedding conditions, similar to B6 mouse pups.

This is in contrast to what was expected, considering that BTBR mice display behavioral phenotypes with relevance to all diagnostic core symptoms of ASD (for review see: Blanchard et al., 2012; Meyza et al., 2013; Careaga et al., 2015). The present findings are further in contrast to a study by Yang et al. (2012a) who reported that the strain of the partner during reciprocal social interactions has minimal effects on the social behavioral repertoire displayed by BTBR mice, suggesting that adult BTBR mice have difficulties in adjusting their behavior to different social contexts.

The fact that emission of isolation-induced USV is affected by social context in BTBR mouse pups might be viewed as a challenge for the BTBR inbred strain of mice as a mouse model for ASD. However, it has to be emphasized that very little evidence is available up to now supporting the notion that the inhibition of isolation-induced USV caused by the presence of odors from mothers and littermates allows the reliable assessment of ASD-relevant behavioral alterations in mouse pups. Probably the strongest finding in support of such a notion was reported by Moles et al. (2004). They showed that μ-opioid deficient mice do not display a reduction in isolation-induced USV emission rates when tested under social odor conditions, whereas in wildtype controls a clear reduction was evident. The lack of a calming response in μ-opioid deficient mice is consistent with a variety of other social and communication deficits displayed by this ASD mouse model (Tian et al., 1997; Wöhr et al., 2011a; Cinque et al., 2012; Becker et al., 2014; Gigliucci et al., 2014; for review see: Oddi et al., 2013).

It has further to be emphasized that also little is known about the general mechanisms underlying the inhibition of isolation-induced USV caused by the presence of odors from mothers and littermates in mouse pups. In a pioneering study by Branchi et al. (1998), comparing three different social odor contexts, namely clean bedding material, bedding material from the home cage, and bedding material from a male cage, CD-1 mice tended to vocalize less in the latter two social odor contexts. In similar studies by Marchlewska-Koj et al. (1999) and Kapusta and Szentgyörgyi (2004), CBA mouse pups emitted shorter isolation-induced USV when tested under home cage bedding conditions as compared to clean bedding conditions, while isolation-induced USV emission rates and peak frequency were not affected. Finally, Lemasson et al. (2005) reported no effect of home cage odor on isolation-induced USV emission rates in B6 mice. In comparison to most available studies, the inhibition reported in the present study appears therefore to be comparatively strong, which is particularly surprising in case of the BTBR mouse model for ASD. One of the possible reasons for the comparatively strong odor effects is that isolation-induced USV were recorded for 5 min, whereas relatively short recording durations were used in most other studies (Branchi et al., 1998; Marchlewska-Koj et al., 1999; Kapusta and Szentgyörgyi, 2004; Lemasson et al., 2005). However, the detailed time course analysis speaks against this idea, as the inhibition of isolation-induced USV in mouse pups tested under soiled bedding conditions was most prominent in the first few minutes of testing, particularly in BTBR mouse pups.

While BTBR mouse pups unexpectedly displayed a calming response and emitted fewer isolation-induced USV when tested under soiled as compared to clean bedding conditions, social odor context had no effect on acoustic call features, such as call duration, peak frequency, peak amplitude, and frequency modulation, in BTBR mouse pups. This is in stark contrast to what was seen in B6 mouse pups, which emitted isolation-induced USV with shorter call durations and lower levels of frequency modulation under soiled bedding conditions as compared to clean bedding conditions.

At least three possible mechanisms for the reduced adjustment to different social contexts in BTBR mouse pups can be considered. Firstly, the fact that social odor context had no effect on acoustic call features in BTBR mouse pups could be due to deficits in detecting changes in the social context caused by olfactory impairments. However, BTBR mice displayed normal olfactory abilities, both in non-social test paradigms, such as the buried food task (Moy et al., 2007), as well as in social ones, like the preference for social novelty task (Moy et al., 2007; McFarlane et al., 2008). Consistently, olfactory habituation/dishabituation in response to a sequence of non-social and social odors was evident in BTBR mice, yet it was clearly less prominent than in B6 mice (Yang et al., 2012a). It was further reported that exploratory behavior displayed by BTBR mice in the hole board task can be altered by presenting soiled bedding (Moy et al., 2008). Moreover, in a recent study on female-induced USV and scent marking behavior in adult male mice, both, BTBR and B6 males, spent a similar amount of time in proximity to a salient olfactory social cue, a spot of female urine, indicating that female urine evoked similar levels of interest in BTBR and B6 males (Wöhr et al., 2011b). Also in pups evidence for intact social olfactory abilities was provided. Specifically, in the homing test, in which mouse pups are exposed to clean bedding on one side and soiled bedding from the home cage on the other, it took BTBR mouse pups less time to reach the side containing the soiled bedding than B6 mouse pups, yet the finding is difficult to interpret due to an overall increased level of locomotor activity in BTBR mouse pups (Scattoni et al., 2008). Finally, the present results show that both, BTBR and B6 mouse pups, display a calming response and emit fewer isolation-induced USV when tested under soiled as compared to clean bedding conditions. Together, this supports the interpretation that BTBR mice are able to process social olfactory cues, both in infancy and adulthood, indicating that the observed deficit in behavioral adjustment to different social contexts in BTBR mouse pups is not due to olfactory impairments.

Secondly, a limited ability to adjust to different social contexts could be the reason for the fact that social odor context had no effect on acoustic call features in BTBR mouse pups. The unusual repertoire of USV categories seen in BTBR mouse pups, including high levels of harmonics, two-syllable, and composite calls (Scattoni et al., 2008), possibly speaks for a limited ability of BTBR mouse pups to modulate the acoustic call features of isolation-induced USV, yet the richness of USV subtypes (Scattoni et al., 2008) and call clusters in the present study speaks against it.

Thirdly, a reduced motivation to adjust to different social contexts could also be the reason and on the basis of the data available it is currently not possible to differentiate between the two possible mechanisms. In support of the latter mechanism it was shown that BTBR mice are characterized by a reduction in social motivation (Pearson et al., 2012; Martin et al., 2014). Specifically, Pearson et al. (2012) found no evidence for social conditioned place preference in BTBR but in B6 mice. Likewise, Martin et al. (2014) reported that BTBR mice had lower breaking points than B6 mice when lever pressing for a social reward, namely access to a conspecific. Yet, it has to be mentioned that breaking points for food reward were also lower in BTBR, questioning the specificity of the motivational deficit for the social domain. Finally, it has to be emphasized that a reduced motivation to adjust to different social context could also be due to altered levels of anxiety, with anxiety levels being possibly elevated in BTBR mice (Benno et al., 2009; Frye and Llaneza, 2010; Pobbe et al., 2011; Gould et al., 2014; Langley et al., 2015).

In line with the findings obtained by Scattoni et al. (2008) and Schwartzer et al. (2013), BTBR mouse pups emitted more isolation-induced USV than B6 mouse pups in the present study. It is currently unclear what is causing this strain difference in isolation-induced USV emission rates. It is tempting to speculate that the strain effect is due to the marked difference in body weight and/or size between BTBR and B6. Yet, Scattoni et al. (2008) showed that FVB/NJ mouse pups vocalized almost as little as B6 mouse pups, despite being close to BTBR mouse pups in body weight. What also appears possible is that the strain effect is caused by a difference in anxiety-related behavior. Isolation-induced USV have been repeatedly associated with anxiety in various behavioral studies (for review see: Schwarting and Wöhr, 2012). For instance, mice selectively bred for high anxiety-related behavior on the elevated plus maze emit more isolation-induced USV as pups than mice selectively bred for low anxiety levels (Krömer et al., 2005; Frank et al., 2009); a finding confirmed and extended by Kessler et al. (2011) who showed that this difference is not affected by cross-fostering and thus likely reflects a line-dependent change in innate anxiety. Also pharmacological studies support this view (for review see: Miczek et al., 1995). For instance, anxiolytic benzodiazepines and other positive modulators of GABA receptors inhibit isolation-induced USV in mouse pups (Benton and Nastiti, 1988; Nastiti et al., 1991; Cirulli et al., 1994; Fish et al., 2000; Takahashi et al., 2009). High levels of isolation-induced USV in BTBR mouse pups could hence reflect higher responses to stress or higher levels of anxiety-like traits. In fact, Schwartzer et al. (2013) found that the already high isolation-induced USV emission rates in BTBR mouse pups can be further enhanced by Poly I:C exposure during pregnancy, mimicking a viral infection and known to increase anxiety-related behavior in adulthood, including higher emission rates of fear-induced USV (Yee et al., 2012). Recently, Langley et al. (2015) further reported increased anxiety-related behavior in juvenile BTBR mice in the elevated plus maze. Moreover, Pobbe et al. (2011) described more defensiveness to animate threat stimuli, such as a predator, and an inconsistent response pattern in elevated plus maze and zero maze in adult BTBR mice. Most studies, however, did not report an anxiety-like phenotype in adult BTBR mice in standard tasks, including elevated plus-maze, zero maze, and light-dark box (Moy et al., 2007; McFarlane et al., 2008; Benno et al., 2009; Yang et al., 2009; Silverman et al., 2010b; Chadman, 2011; Molenhuis et al., 2014). Also, significantly higher plasma corticosterone levels and exaggerated responses to stress were repeatedly reported in juvenile and adult BTBR mice (Benno et al., 2009; Frye and Llaneza, 2010; Gould et al., 2014), yet no evidence for an abnormal stress response was detected by Silverman et al. (2010b) in adulthood. Thus, it is not clear whether high levels of isolation-induced USV in BTBR mouse pups reflect higher responses to stress or higher levels of anxiety-like traits and future studies on anxiety-related behavior in infant and juvenile BTBR mice appear indicated. Finally, it is also not clear whether strain differences in anxiety-like behavior and the production of isolation-induced USV are due to differences in maternal behavior. Yang et al. (2007b) reported typical maternal caregiving behavior in BTBR females and no major changes in the behavioral repertoire of BTBR offspring were seen following cross-fostering to B6 females. However, when BTBR embryos were transferred to B6 females, significant improvements in social and repetitive behavior were observed, yet anxiety-like behavior and isolation-induced USV were not assessed and it is unclear whether the observed changes are due to differences in the maternal immune environment or social factors (Zhang et al., 2013).

It is further in line with the findings obtained by Scattoni et al. (2008) that the isolation-induced USV emitted by BTBR mouse pups were longer in call duration, lower in peak frequency, but higher in peak amplitude than the ones emitted by B6 mouse pups. The higher level of frequency modulation of isolation-induced USV emitted by BTBR muse pups observed in the present study is probably reflecting the larger proportion of harmonics, two-syllable, and composite calls, as reported by Scattoni et al. (2008) before. The present findings show that these strain differences are robust and reliably detectable in two different social odor contexts, namely clean and soiled bedding conditions. Such strain differences might again be due to differences in body weight and/or size, but also related characteristics, including the length of their vocal cords.

The present study further identified clusters of isolation-induced USV emitted by BTBR and B6 mouse pups under clean and soiled bedding conditions by means of density plots. In BTBR mouse pups, four prominent call clusters were detected, virtually independent from social odor context, further highlighting their limited ability and/or reduced motivation to adjust to different social contexts. One cluster was characterized by short call durations and low levels of frequency modulation. The other three clusters were characterized by long call durations, with varying levels of frequency modulation, namely low, moderate, and high. Therefore, by means of the quantitative approach applied here, no clear evidence for the existence of 10 distinct USV subtypes as reported by Scattoni et al. (2008) was obtained. However, it has to be emphasized that the 10 USV subtypes differentiated by Scattoni et al. (2008) were identified by means of visual analyses of waveform patterns; a strategy that allows to take various different call features into account, while the quantitative approach applied here is based on two factors only, namely call duration and frequency modulation. Yet, despite the different approach and the difference in USV subtypes/clusters, remarkable consistencies were obtained. For instance, Scattoni et al. (2008) reported that USV subtypes characterized by a high level of frequency-modulation, such as harmonics, two-syllable, and frequency step calls, are longer in duration than less frequency-modulated USV subtypes. This is perfectly in line with the present findings obtained by means of density plots. Future studies are needed to test whether certain USV subtypes reported by Scattoni et al. (2008) are exclusively present in specific call clusters.

Call clustering markedly differed between BTBR and B6 mouse pups. While in BTBR four prominent call clusters were detected, only two prominent call clusters were detected in B6 mouse pups. One cluster was characterized by short call durations and low levels of frequency modulation, whereas the second one was characterized by comparably long call durations and moderate levels of frequency modulation. Interestingly, in B6 mouse pups, social odor context affected call clustering, with the second call cluster characterized by comparably long call durations and moderate levels of frequency modulation being less prominent and coherent, in line with the overall reduced mean call duration and frequency modulation. Shorter call durations in mouse pups tested in soiled bedding were reported before (Marchlewska-Koj et al., 1999; Kapusta and Szentgyörgyi, 2004).

Finally, an additional sequential analysis of the durations of subsequent isolation-induced USV indicated that the USV emission pattern is not random in BTBR mouse pups tested under both, clean and soiled bedding conditions, and that the temporal pattern does not differ significantly from the one obtained in B6 mouse pups. Specifically, in both, BTBR and B6 mouse pups, the durations of given isolation-induced USV could be predicted by the durations of the previous ones. Considering the unusual pattern of USV categories displayed by BTBR mouse pups (Scattoni et al., 2008), it might seem surprising that the temporal organization as assessed here appears to be unaltered in the BTBR mouse model for ASD, particularly because a distorted sequential organization was recently reported in a genetic mouse model for ASD, the *Shank1* deficient mouse (Wöhr, 2014). *Shank1* deficient mice display a variety of behavioral alterations with relevance to ASD (Hung et al., 2008; Silverman et al., 2011; Wöhr et al., 2011b; Sungur et al., 2014; for a USV emission pattern analysis in *Shank2* deficient mice see: Ey et al., 2013).

## Conclusion

In accordance with their behavioral phenotypes with relevance to all diagnostic core symptoms of ASD, it was predicted that BTBR mouse pups would not display a calming response when tested under soiled bedding conditions with home cage bedding material containing maternal odors, and that similar isolation-induced USV emission rates would be seen in BTBR mice tested under clean and soiled bedding conditions. Unexpectedly, however, the present findings show that BTBR mouse pups display such a calming response and emit fewer isolation-induced USV when tested under soiled as compared to clean bedding conditions, similar to B6 mouse pups. Yet, in contrast to B6 mouse pups, which emitted isolation-induced USV with shorter call durations and lower levels of frequency modulation under soiled bedding conditions, social odor context had no effect on acoustic call features in BTBR mouse pups. This indicates that the BTBR mouse model for ASD does not display deficits in detecting changes in social context, but has a limited ability and/or reduced motivation to adjust to them.

### Conflict of interest statement

The author declares that the research was conducted in the absence of any commercial or financial relationships that could be construed as a potential conflict of interest.
